# EHPnet: WHO Ultraviolet Radiation Website

**Published:** 2008-04

**Authors:** Erin E. Dooley

**http://www.who.int/uv/en/**

The World Health Organization (WHO) has devoted a section of its website to activities related to the health effects of sunlight. The homepage for this section provides a brief overview of the topic along with links to resources such as the 2006 report *Solar Ultraviolet Radiation: Global Burden of Disease from Solar Ultraviolet Radiation* and a brochure on how to enjoy sunlight safely.

A Frequently Asked Questions section contains information on topics including ultraviolet radiation (UVR), tanning beds, and the UV Index. The site also has subsections on the health effects of UVR and on sun protection. The health effects page discusses the beneficial health effects of UVR, such as how it helps the body produce vitamin D and acts in the treatment of psoriasis, eczema, and jaundice. Other information addresses the relationship between sun exposure and skin cancer, blindness, and cell-mediated immunity. The Sun Protection page lists precautions that can help prevent sun damage and explains how children are more vulnerable to the health threats posed by too much exposure to the sun.

The homepage also provides a link to information on the INTERSUN Programme. INTERSUN was established during the 1992 United Nations Conference on Environment and Development.The program has three primary goals: to serve as a source of practical and scientific information relating to the health impact and environmental effects of UVR exposure; to encourage governmental action to reduce UVR-related health problems; and to provide guidance to governments and agencies about establishing effective sun awareness programs. Two areas of special activity are tourism and occupational health. On the Tourism page, INTERSUN provides guidance to tourists and tour operators to help mitigate the impact of short, intense bursts of sun exposure, which are associated with malignant melanoma. The Occupational Health page provides information for arc welders, lab technicians, outdoor workers, and others who encounter high UVR exposures on the job.

